# Properties of the lunar gravity assisted transfers from LEO to the retrograde-GEO

**DOI:** 10.1038/s41598-021-98231-1

**Published:** 2021-09-22

**Authors:** Bo-yong He, Peng-bin Ma, Heng-nian Li

**Affiliations:** State Key Laboratory of Astronautic Dynamics (ADL), Xi’an Satellite Control Center, Xi’an, 710043 China

**Keywords:** Planetary science, Space physics, Astronomy and planetary science, Engineering, Mathematics and computing

## Abstract

The retrograde geostationary earth orbit (retro-GEO) is an Earth’s orbit. It has almost the same orbital altitude with that of a GEO, but an inclination of 180°. A retro-GEO monitor-satellite gives the GEO-assets vicinity space-debris warnings per 12 h. For various reasons, the westward launch direction is not compatible or economical. Thereby the transfer from a low earth orbit (LEO) to the retro-GEO via once lunar swing-by is a priority. The monitor-satellite departures from LEO and inserts into the retro-GEO both using only one tangential maneuver, in this paper, its transfer’s property is investigated. The existence of this transfer is verified firstly in the planar circular restricted three-body problem (CR3BP) model based on the Poincaré-section methodology. Then, the two-impulse values and the perilune altitudes are computed with different transfer durations in the planar CR3BP. Their dispersions are compared with different Sun azimuths in the planar bi-circular restricted four-body problem (BR4BP) model. Besides, the transfer’s inclination changeable capacity via lunar swing-by and the Sun-perturbed inclination changeable capacity are investigated. The results show that the two-impulse fuel-optimal transfer has the duration of 1.76 TU (i.e., 7.65 days) with the minimum values of 4.251 km s^−1^ in planar CR3BP, this value has a range of 4.249–4.252 km s^−1^ due to different Sun azimuths in planar BR4BP. Its perilune altitude changes from 552.6 to 621.9 km. In the spatial CR3BP, if the transfer duration is more than or equal to 4.00 TU (i.e., 17.59 days), the lunar gravity assisted transfer could insert the retro-GEO with any inclination. In the spatial BR4BP, the Sun’s perturbation does not affect this conclusion in most cases.

## Introduction

**Figure 1:**
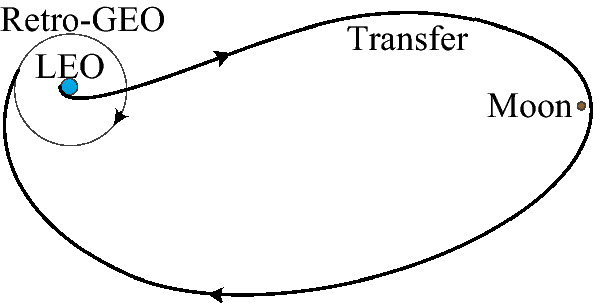
The transfer from LEO to the retrograde-GEO via lunar swing-by.

As we known, the geostationary earth orbit (GEO) has the same period as the Earth's rotation period. Its sub-point coverage is almost still. Many important satellites for navigation, remote sensing, data-relay, meteorology, ocean monitoring and land-resources monitoring are deployed on the GEO. For decades, due to the exponential growth of the number of the GEO satellites, the rocket terminal stage, the failed-satellites, the space debris, and the safety box limitation to accommodate perturbation, many important GEO positions deploys satellites under collocation strategy^1^. The circumstance of the GEO-assets is serious about the quite crowded GEO-belt. On July 28, 2014 and August 19, 2016, the USA successfully launched four GEO satellites, GSSAP-1/2 and GSSAP-3/4 (Geosynchronous space Situational Awareness-Ness Program), respectively^2^. They are possible to give a few of these GEO-assets vicinity early debris-warning by raising or lowering their orbital altitudes, but their orbit maneuver fuel-cost greatly limits their patrol-range. The retrograde GEO (retro-GEO) is an earth’s orbit, which has almost the same orbital altitude with that of a GEO, but an inclination of 180°. A monitor-satellite on the retro-GEO gives all of the GEO-assets vicinity debris-warning per 12 h. The transfer from a low earth orbit (LEO) to the retro-GEO via once lunar swing-by is shown in Fig. 1. This lunar gravity assisted manner avoids the difficulty that the number of the ground-measurement and tracks facilities is a few for westward-launch directly. In respect that the usual space launch rocket has an eastward-launch scenarios, in this way, it saves the launching energy-cost via the Earth’s rotation force.

The dynamics description of the lunar swing-by orbit could date back to *Issac Newton* in 1687. The circumlunar free-return orbit in Apollo mission is a famous practical activity to improve the safety of the crews in the manned space missions^3^. The probe *Hiten* launched by Japan in 1990 acted a double lunar swing-by space-flight^4^. In 1998, Hughes saved the original ISEE-3/ICE via multi-lunar swing-by and multi-maneuvers. It becomes the first successful space legend of saving satellites^5^. Zeng et al.^6^ studied the lunar swing-by transfer using a simplified double two-body hypothesis orbital model, the transfer launches up from a high-latitude launch-pad and transfers to the retro-GEO. His result shows that the transfer via lunar swing-by saves maneuver fuel-cost, but the orbital perigee altitude and the inclination during the return retro-GEO phase in his work did not match the retro-GEO. Luo et al.^7^ described the mechanics of the double-lunar swing-by, expatiated the sensitive property of the trajectories for deep-space exploration via lunar swing-by to a certain extent.

As the number of the GEO satellites grows exponentially, the GEO-assets’ safety problem caused by the abandoned-satellites and space-debris is becoming more and more serious^8^. In fact, Oberg^9^ presented the pioneering retro-GEO concept as early as 1984, and explained the flight-manner saves fuel-cost to deploy a satellite on the retro-GEO via lunar swing-by. Kawase et al.^10,11^ advanced the reasonable proposal that a monitor-satellite on the retro-GEO plays the debris-warning alertor for all of the GEO-assets. Aravind et al.^12^ compared the same satellite’s left fuel by several different typical-flight sceneries from an LEO to the final retro-GEO. Aravind also tried to calculate the final left-fuel using lunar swing-by to compare with the typical-flight sceneries, but the orbital perigee altitude during the return retro-GEO phase is 124.75 km, the value is far below the desired retro-GEO orbital altitude.

To sum up, the retro-GEO monitor-satellite gives all of the GEO-assets vicinity debris-warning per 12 h, and the flight-manner via lunar swing-by saves fuel-cost. But, the analysis of examples until now in references^6,12^ did not satisfy the orbital element constraints. In this paper, the purpose is to discover the fundamental properties of the transfers in the classical circular restricted three-body problem (CR3BP) model and the bi-circular four-body problem (BR4BP) model, such as, whether the transfer via lunar swing-by with the orbital element constraints (i.e., Its orbital altitude and inclination during the departure LEO and insert retro-GEO phase) and the two-impulse tangential maneuvers is existed or not? What is the foundational feature of this transfer in the planar model? How much is the lunar gravity assisted effect for the orbital inclination changeable capacity? After the concise statement of the problem in “Problem statement”, the first two questions are exhibited in “Properties in the planar model”, and the last question is exhibited in “Properties in the spatial model”. The paper ends with “Conclusions” which gives some brief conclusions and implications on this topic.

## Problem statement

### Advantages via lunar swing-by

If the retro-GEO satellite is deployed directly by the westward-launch manner for China, there are two problems. First, the most satellites of China launched up via an eastward-launch manner, there is no conventional landing area for the first and second stage-debris of the westward-launch rockets. The sub-points of the first and second stage-debris of the westward-launch rocket may spread to the densely populated area and even out of border. Second, the eastward-launch manner can be accurately measured and controlled by the mature ground stations of *China Xi’an Satellite Control Center*, while the westward-launch cannot be supported by mature ground stations. Moreover, the equatorial radius of the Earth is about 6378.134 km, and its rotation angular velocity is about 7.292115 × $10^{ - 5}$ rad s^−1^. The beneficial velocity-increment of the eastward-launch manner launched-up from the equator is about 465 m s^−1^, while there is about additional 465 m s^−1^ velocity-increment needs to be overcome by the westward-launch manner. It is an obvious contrast to the two manners’ fuel-cost.

The moon is the Earth's sole natural celestial body and is the most man-made probes visited celestial body by-far. In particular, the Chang'E-series lunar probes of China show that China has mastered the techniques of the lunar probes launch-up, precise orbit determination, and orbital control around the Moon^13^.

### Orbital dynamics and constraints

**Figure 2:**
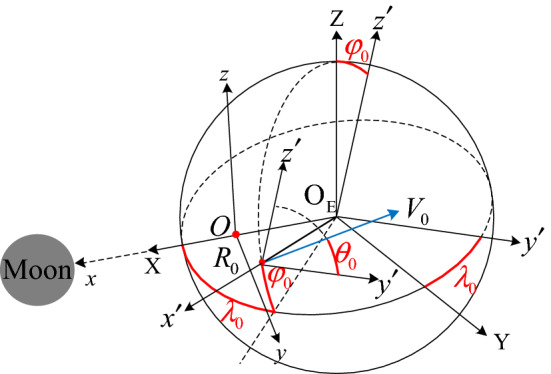
The vectors of the position and velocity at the epoch of trans-lunar injection.

The most classical orbital model of describing a probe’s path in the Earth-Moon space is the CR3BP model. In CR3BP, there are two primary bodies $\left[ {P_{1} ,P_{2} } \right]$ and the probe $P$ of masses $m_{1} > m_{2} \gg m$, respectively. The motion of $\left[ {P_{1} ,P_{2} } \right]$ is not affected by the probe $P$, and move around their common center of mass under their mutual gravity. In the Earth-Moon space, $\left[ {P_{1} ,P_{2} } \right]$ represent the Earth and the Moon, respectively. Let $\mu = {{m_{2} } \mathord{\left/ {\vphantom {{m_{2} } {\left( {m_{1} + m_{2} } \right)}}} \right. \kern-\nulldelimiterspace} {\left( {m_{1} + m_{2} } \right)}}$ denotes the mass ratio of $P_{2}$ to the total mass. The motion of the probe $P$ relative to a co-rotating coordinate system $O{ - }xyz$ as shown in Fig. 2 with the origin at their common center, and in normalized distance, mass, time units, and speed unit are listed in Table 1.

**Table 1:** Earth-moon space constants^14^.

Symbol	Value	Units	Meaning
$\mu$	1.21506683 × 10^–2^	–	The mass ratio of the moon to the earth
$m_{s}$	3.28900541 × 10^5^	–	Scaled mass of the sun
$\rho$	3.88811143 × 10^2^	–	Scaled Sun–(earth + moon) distance
$\omega_{s}$	− 9.25195985 × 10^–1^	–	Scaled angular velocity of the sun
$l_{em}$	3.84405000 × 10^8^	m	Earth–moon distance
$\omega_{em}$	2.66186135 × 10^–6^	s^−1^	Earth–moon angular velocity
$R_{0}$	6378	km	Mean earth’s radius
$R_{{\text{f}}}$	1738	km	Mean moon’s radius
$h_{{0}}$	167	km	Altitude of departure orbit
$h_{{\text{f}}}$	35,786	km	Altitude of arrival orbit
$DU$	3.84405000 × 10^8^	m	Distance unit
$TU$	4.34811305	days	Time unit
$VU$	1.02323281 × 10^3^	m s^−1^	Velocity unit

The differential equation description of CR3BP^15^ is

1 $$\ddot{x} - 2\dot{y} = \frac{{\partial \Omega_{3} }}{\partial x},\ddot{y} + 2\dot{x} = \frac{{\partial \Omega_{3} }}{\partial y},\ddot{z} = \frac{{\partial \Omega_{3} }}{\partial z}.$$

Here, the effective potential function is

2 $$\Omega_{3} = \frac{1}{2}\left( {x^{2} + y^{2} + z^{2} } \right) + \frac{1 - \mu }{{r_{1} }} + \frac{\mu }{{r_{2} }} + \frac{1}{2}\mu \left( {1 + \mu } \right),$$

with $r_{1} = \sqrt {\left( {x + \mu } \right)^{2} + y^{2} + z^{2} }$,$r_{2} = \sqrt {\left( {x + \mu - 1} \right)^{2} + y^{2} + z^{2} }$ in (2). $\left[ {P_{1} ,P_{2} } \right]$ are located at $\left( { - \mu ,0,0} \right)$, $\left( {1 - \mu ,0,0} \right)$, respectively.

Select the moment of the trans-lunar injection (i.e., the subscript ‘0’ means the start epoch) as the epoch of the Earth-centered instantaneous inertial coordinate system ${\text{O}}_{{\text{E}}} {\text{ - XYZ}}$, ${\text{O}}_{{\text{E}}} {\text{ - X}}$ points to the center of the Moon, ${\text{O}}_{{\text{E}}} {\text{ - Z}}$ points to the angular-momentum direction of the Moon’s path, ${\text{O}}_{{\text{E}}} {\text{ - Y}}$ constructs the Cartesian coordinate system with the other two axis. In the frame of ${\text{O}}_{{\text{E}}} {\text{ - XYZ}}$, the position and velocity vectors of $\left[ {{\varvec{R}}_{0} ,{\varvec{V}}_{0} } \right]$ are described as (3).

3 $$\left\{ \begin{gathered} {\varvec{R}}_{0} = R_{0} \cdot {\varvec{M}}_{{\text{z}}} \left( { - \lambda_{0} } \right){\varvec{M}}_{{\text{y}}} \left( {\varphi_{0} } \right) \cdot \left[ {\begin{array}{*{20}l} 1 & 0 & 0 \\ \end{array} } \right]^{{\text{T}}} \hfill \\ {\varvec{V}}_{0} = V_{0} \cdot {\varvec{M}}_{{\text{z}}} \left( { - \lambda_{0} } \right){\varvec{M}}_{{\text{y}}} \left( {\varphi_{0} } \right) \cdot \left[ {\begin{array}{*{20}l} 0 & {\cos \theta_{0} } & {\sin \theta_{0} } \\ \end{array} } \right]^{{\text{T}}} . \hfill \\ \end{gathered} \right.$$

Here, $R_{0}$ and $V_{0}$ describe the values of the position and velocity, respectively. $\lambda_{0}$ and $\varphi_{0}$ describe the position vector. $\theta_{0}$ describes the direction of the velocity vector in the plane of the position vector. ${\varvec{M}}_{{\text{y}}}$ and ${\varvec{M}}_{{\text{z}}}$ are the fundamental coordinate transformation matrixes, the other one ${\varvec{M}}_{{\text{x}}}$ does not be used here. The obvious constraint is satisfied as ${\text{dot}}\left( {{\varvec{R}}_{0} ,{\varvec{V}}_{0} } \right) = 0$ means that the trans-lunar injection impulse is tangential to the position vector at that epoch.

In CR3BP, $O$ denotes the origin of the co-rotating coordinate system $O{ - }xyz$. $O{ - }x$ follows the direction of the Moon’s center. $O{ - }z$ follows the angular-momentum direction of the Moon’s path. $O{ - }y$ constructs the active Cartesian coordinate system with the other two axes. The vectors of the position and velocity in $O{ - }xyz$ have constraints as (4).

4 $$\left\{ \begin{gathered} {\varvec{R}}_{0} = \user2{r}_{0} + \left[ {\begin{array}{*{20}l} \mu & 0 & 0 \\ \end{array} } \right]^{{\text{T}}} \hfill \\ {\varvec{V}}_{0} = {\varvec{v}}_{0} \user2{ + \omega } \times {\varvec{r}}_{0} . \hfill \\ \end{gathered} \right.$$

Here, ${\varvec{r}}_{0} = \left[ {x,y,z} \right]_{0}^{{\text{T}}}$, ${\varvec{v}}_{0} = \left[ {\dot{x},\dot{y},\dot{z}} \right]_{0}^{{\text{T}}}$, ${\varvec{\omega}} = \left[ {\omega_{{\text{x}}} ,\omega_{{\text{y}}} ,\omega_{{\text{z}}} } \right]^{{\text{T}}} = \left[ {0,0,1} \right]^{{\text{T}}}$. And the additional rotation velocity is

5 $${\varvec{\omega}} \times {\varvec{r}}_{0} = \left[ {\begin{array}{*{20}c} 0 & { - \omega_{{\text{z}}} } & {\omega_{{\text{y}}} } \\ {\omega_{{\text{z}}} } & 0 & { - \omega_{{\text{x}}} } \\ { - \omega_{{\text{y}}} } & {\omega_{{\text{x}}} } & 0 \\ \end{array} } \right]{\varvec{r}}_{0} .$$

Select the epoch of the perigee during the return retro-GEO phase as the final moment (i.e., the subscript ‘f’ means the final). The position vector and the velocity vector satisfy the constraint of ${\text{dot}}\left( {{\varvec{R}}_{{\text{f}}} ,{\varvec{V}}_{{\text{f}}} } \right) = 0$ at this epoch. Besides, another constraint is that the perilune altitude of the transfer is more than zero at least.

A more precise orbit model is the BR4BP model. BR4BP considers the four-body $P_{3}$ base on CR3BP. In the Earth–Moon space, $P_{3}$ denotes the Sun. The Sun-perturbed orbital dynamics model of $P$^16^ is

6 $$\ddot{x} - 2\dot{y} = \frac{{\partial \Omega_{4} }}{\partial x},\ddot{y} + 2\dot{x} = \frac{{\partial \Omega_{4} }}{\partial y},\ddot{z} = \frac{{\partial \Omega_{4} }}{\partial z}.$$

The moon orbit inclination on the ecliptic is about 5°, the planar BR4BP catches basic insights of the real four-body dynamics^17^. The equivalent potential function is

7 $$\Omega_{4} \left( {x,y,z,t} \right) = \Omega_{3} \left( {x,y,z} \right) + \frac{{m_{s} }}{{r_{3} \left( t \right)}} - \frac{{m_{s} }}{{\rho^{2} }}\left( {x\cos \left( {\omega_{s} t} \right) + y\sin \left( {\omega_{s} t} \right)} \right).$$

Here, $m_{s}$ denotes the scaled mass of the Sun. $\rho$ denotes the scaled the Sun and the Earth–Moon barycenter distance. $\omega_{s}$ denotes the angular velocity of the Sun in the Earth–Moon rotating frame. The phase angle of the Sun to the $O - x$ axis is $\omega_{s} t$ at the moment. Hence the position of the Sun is $\left( {\rho \cos \left( {\omega_{s} t} \right),\rho \sin \left( {\omega_{s} t} \right)} \right)$. The distance from the lunar probe $P$ to the Sun is

8 $$r_{3} \left( t \right) = \sqrt {\left( {x - \rho \cos \left( {\omega_{s} t} \right)} \right)^{2} + \left( {y - \rho \sin \left( {\omega_{s} t} \right)} \right)^{2} } .$$

## Properties in the planar model

### Existence verification

Considering that the transfers solved by the previous works^6,12^ did not completely satisfy the constraints (i.e., two tangential maneuvers and the perigee altitude during the return retro-GEO phase), its existence need to be firstly verified. Poincaré proves that there is no general analytical mathematical procedure of computing the transfers in the three-body problem. He suggested the Poincaré section methodology to formulate the property of the multidimensional nonlinear differential equations. This methodology gives a clear criterion and qualitative conclusion for these problems. The planar CR3BP is a dimension-reduced result of (1), the $O - z$ direction is decoupled from the other two directions in $O - xyz$. To be specific, the orbital element $\left[ {\varphi ,\theta } \right]$ are zero in the full time. In this paper, it is used flexibly to obtain the transfer’s existence features.

**Figure 3:**
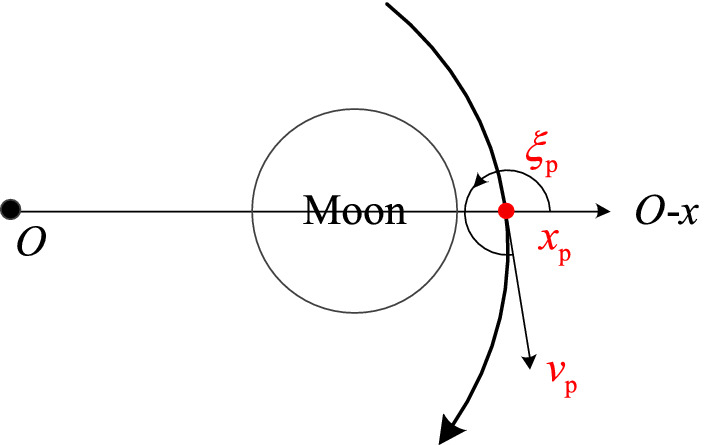
The select Poincaré section.

Select the orbital elements both at the moment of trans-lunar injection and at the moment of the perigee during the return retro-GEO phase as the traversal searching variables. The trans-lunar phase is computed using the numerical integration by the positive direction of time, while the return retro-GEO phase is computed using the numerical integration by the negative direction of time. Both stop at the Poincaré section. The Poincaré section is selected here at the axis of $O - x$ and is far-side from the Earth and the Moon. It has three orbital elements as shown in Fig. 3, the position on $O - x$ of $x_{{\text{p}}}$, the velocity value of $v_{{\text{p}}}$, and the velocity angle of $\xi_{{\text{p}}}$. All of them is at the epoch when the position component of $O - y$ is zero (i.e., $y_{{\text{p}}} = 0$). The subscript ‘p’ denotes this moment just because it is close to the perilune epoch.

**Figure 4:**
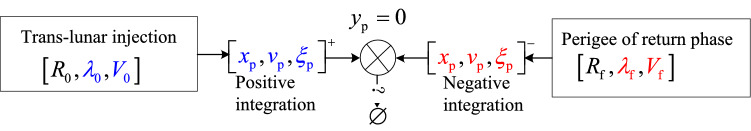
Illustration of the existence validation strategy.

The geocentric distance $R_{0}$ at the epoch of trans-lunar injection is a constant, because it is dominated by the capability of the rocket. The geocentric distance $R_{{\text{f}}}$ at the moment of the perigee during the return retro-GEO phase is the same with that of the GEO. This transfer’s existence validation converts into an appointment at the selected Poincaré section as expressed in Fig. 4.

The orbital elements $\left[ {x_{{\text{p}}} ,v_{{\text{p}}} ,\xi_{{\text{p}}} } \right]^{ + }$ and $\left[ {x_{{\text{p}}} ,v_{{\text{p}}} ,\xi_{{\text{p}}} } \right]^{ - }$, which are computed from $\left[ {R_{0} ,\lambda_{0} ,V_{0} } \right]$ and $\left[ {R_{{\text{f}}} ,\lambda_{{\text{f}}} ,V_{{\text{f}}} } \right]$, respectively, meet at the Poincaré section of $y_{{\text{p}}} = 0$. If there is a non-empty intersection set about $\left[ {x_{{\text{p}}} ,v_{{\text{p}}} ,\xi_{{\text{p}}} } \right]^{ + }$ and $\left[ {x_{{\text{p}}} ,v_{{\text{p}}} ,\xi_{{\text{p}}} } \right]^{ - }$, this transfer’s existence is sufficiently validated. Otherwise, there is no transfer satisfied the constraints in “Orbital dynamics and constraints” in the planar CR3BP.

**Figure 5:**
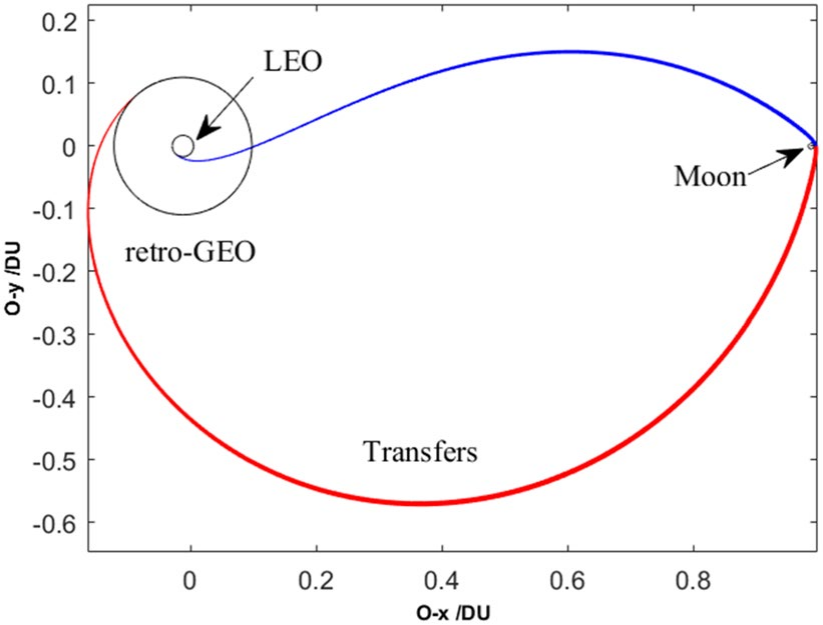
The planar trajectories of the transfers.

**Figure 6:**
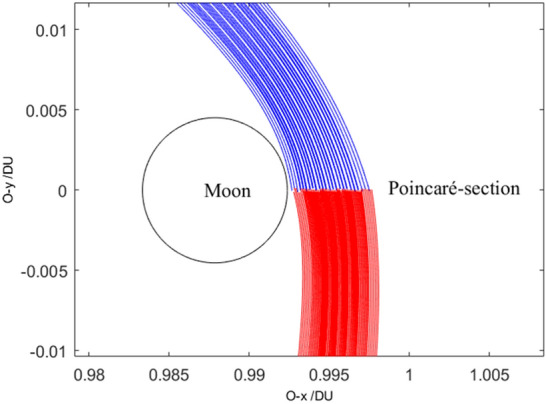
The partial enlarged detail of the Poincaré-section.

**Figure 7:**
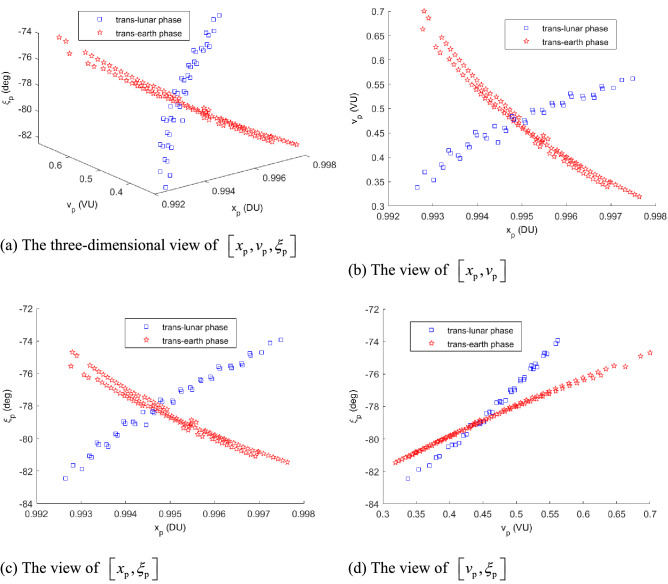
The three-dimensional parameter of the Poincaré section.

Without loss of generality, set $R_{0} =$ 6545 km (i.e., 6378 + 167)^14^, and set $R_{{\text{f}}} =$ 42,164 km (i.e. 6378 + 35,786). The orbital elements of plotting all of the trajectories in Fig. 5 are listed in Table 2. Its partial detail trajectories enlarged of the Poincaré section are plotted in Fig. 6. The Poincaré section’s three plane views are plotted in Fig. 7a–d. The blue square is the trans-lunar phase of $\left[ {x_{{\text{p}}} ,v_{{\text{p}}} ,\xi_{{\text{p}}} } \right]^{ + }$ while the red pentagram is the return retro-GEO phase of $\left[ {x_{{\text{p}}} ,v_{{\text{p}}} ,\xi_{{\text{p}}} } \right]^{ - }$.

**Table 2:** The orbital elements of plotting the Poincaré section.

Computing orbit elements		Results of Poincaré-section map	
Symbol	Value (units)	Symbol	Value (units)
$R_{0}$	6545 (km)	$x_{{\text{p}}}^{ + }$	[0.9926,0.9975] (DU)
$\lambda_{0}$	[225.1, 225.4] (°)	$v_{{\text{p}}}^{ + }$	[0.3377,0.5615] (VU)
$V_{0}$	[10.9838, 10.985] (km s^−1^)	$\xi_{{\text{p}}}^{ + }$	[− 82.4637, −73.9266] (°)
$R_{{\text{f}}}$	42,164 (km)	$x_{{\text{p}}}^{ - }$	[0.9928, 0.9976] (DU)
$\lambda_{{\text{f}}}$	[126.5, 127.3] (°)	$v_{{\text{p}}}^{ - }$	[0.3190, 0.6998] (VU)
$V_{{\text{f}}}$	[4.1261, 4.1271] (km s^−1^)	$\xi_{{\text{p}}}^{ - }$	[− 81.4566, − 74.6909] (°)

It shows clearly that the Poincaré section is a non-empty set, the transfer’s existence is validated. The transfer exists, which is from an LEO of deploying the retro-GEO via lunar swing-by with two tangential maneuvers.

### Optimal two-impulse solution

In the planar CR3BP, the optimal two-impulse solution is the usual computing goal. The continuation theory is practically useful to solve the transfer which has some sensitive design variables or a long duration^14,18^. From the primary computing results of the Poincaré section in “Existence verification”, some solutions give the goal the initial value of the orbital elements. Then, a continuation frame is built. This frame employs the duration of the transfer plays the role of the continuation element as (9) in the outer layer.

9 $${\text{Outer}}\left\{ \begin{array}{l}{\text{for }}\Delta t = \Delta {t_{\min }}:\Delta {t_{{\text{step}}}}:\Delta {t_{\max }}\\{\text{Inner}}{\kern 1pt} {\kern 1pt} \left\{ \begin{array}{l}\user2{x} = \left[ {{\lambda _0},{V_0},{\lambda _{\text{f}}},{V_{\text{f}}}} \right]\\\min {\kern 1pt} {\kern 1pt} {\kern 1pt} {\kern 1pt} J = \Delta v\\{\text{s}}{\text{.}}{\kern 1pt} {\text{t}}{\text{.}}{\kern 1pt} {\kern 1pt} {\kern 1pt} {\kern 1pt} {\kern 1pt} {\kern 1pt} {\kern 1pt} \varepsilon = 0\end{array} \right.\\{\text{end}}\end{array} \right.$$

The step value of $\Delta t_{{{\text{step}}}}$ can be adjusted as needed (e.g., the optimal iteration convergence performance). In the inner layer, there is just a simple constrained optimization model. The sum of the two-impulse of $\Delta v$ is optioned as the minimum objective. Its expression is

10 $$\Delta v = \left( {V_{0} - \sqrt {{{\mu_{{\text{E}}} } \mathord{\left/ {\vphantom {{\mu_{{\text{E}}} } {R_{0} }}} \right. \kern-\nulldelimiterspace} {R_{0} }}} } \right) + \left( {V_{{\text{f}}} - \sqrt {{{\mu_{{\text{E}}} } \mathord{\left/ {\vphantom {{\mu_{{\text{E}}} } {R_{{\text{f}}} }}} \right. \kern-\nulldelimiterspace} {R_{{\text{f}}} }}} } \right).$$

Here, $$\mu_{{\text{E}}} = 398600.44\,{\text{km}}^{3} \,{\text{s}}^{ - 1}$$. To be the same with the design variables in “Existence verification”, $$\left[ {\lambda_{0} ,V_{0} ,\lambda_{{\text{f}}} ,V_{{\text{f}}} } \right]$$ plays the role of the variables. Refer to our previous work experience^18^, slice the total transfer duration into two segments, $$t_{{{\text{mid}}}}$$ denotes the middle epoch. $$\left( {\vec{x}_{{{\text{mid}}}} ,\vec{y}_{{{\text{mid}}}} ,\vec{\dot{x}}_{{{\text{mid}}}} ,\vec{\dot{y}}_{{{\text{mid}}}} } \right)$$ denotes the flight state which is computed from the LEO by the forward-time numerical integration. $$\left( {\mathop{x}\limits^{\leftarrow} _{{{\text{mid}}}} ,\mathop{y}\limits^{\leftarrow} _{{{\text{mid}}}} ,\overset{\lower0.5em\hbox{$\smash{\scriptscriptstyle\leftarrow}$}}{\dot{x}}_{{{\text{mid}}}} ,\overset{\lower0.5em\hbox{$\smash{\scriptscriptstyle\leftarrow}$}}{\dot{y}}_{{{\text{mid}}}} } \right)$$ denotes the flight state which is computed from the retro-GEO by the reverse-time numerical integration. Under ideal condition, $$\left( {\vec{x}_{{{\text{mid}}}} ,\vec{y}_{{{\text{mid}}}} ,\vec{\dot{x}}_{{{\text{mid}}}} ,\vec{\dot{y}}_{{{\text{mid}}}} } \right) \equiv \left( {\mathop{x}\limits^{\leftarrow} _{{{\text{mid}}}} ,\mathop{y}\limits^{\leftarrow} _{{{\text{mid}}}} ,\overset{\lower0.5em\hbox{$\smash{\scriptscriptstyle\leftarrow}$}}{\dot{x}}_{{{\text{mid}}}} ,\overset{\lower0.5em\hbox{$\smash{\scriptscriptstyle\leftarrow}$}}{\dot{y}}_{{{\text{mid}}}} } \right)$$. But small numerical integration error $$\varepsilon$$ exists in the actual numerical calculation. It will be made up by a mid-course correction in the engineering project.

11 $$\varepsilon { = }\left| {\left( {\vec{x}_{{{\text{mid}}}} ,\vec{y}_{{{\text{mid}}}} ,\vec{\dot{x}}_{{{\text{mid}}}} ,\vec{\dot{y}}_{{{\text{mid}}}} } \right) - \left( {\mathop{x}\limits^{\leftarrow} _{{{\text{mid}}}} ,\mathop{y}\limits^{\leftarrow} _{{{\text{mid}}}} ,\overset{\lower0.5em\hbox{$\smash{\scriptscriptstyle\leftarrow}$}}{\dot{x}}_{{{\text{mid}}}} ,\overset{\lower0.5em\hbox{$\smash{\scriptscriptstyle\leftarrow}$}}{\dot{y}}_{{{\text{mid}}}} } \right)} \right|.$$

**Figure 8:**
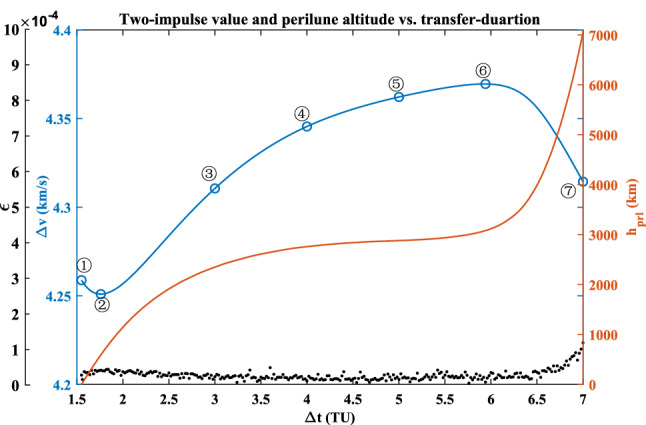
Two-impulse value and perilune altitude vs. transfer-duration.

The sequence quadratic programming (SQP) algorithm in the Matlab *fmincon* function is applied for the inner layer in this paper. Here, the value of $\varepsilon$ is limited to $1 \times 10^{ - 4}$, otherwise, it is considered that the two phases cannot be joined. Addition, another potential constraint is that the value of the perilune altitude $h_{{{\text{prl}}}}$ must be more than zero. The optimal two-impulse solution is shown in Fig. 8.

It can be seen that when the transfer duration approaches to 7 TU, the value of $\varepsilon$ is more than $1 \times 10^{ - 4}$. With the increasing of the transfer duration, the value of the perilune altitude always increases. The optimal two-impulse value, $\Delta v =$ 4.2509 ${\text{km}}\,{\text{s}}^{ - 1}$, occurs at the case that the duration is 1.76 TU (i.e., 7.65 days). While its maximum value, $\Delta v =$ 4.3695 ${\text{km}}\,{\text{s}}^{ - 1}$, occurs at the case that the duration is 5.94 TU (i.e., 25.83 days). The detail elements of the seven solutions marked in Fig. 8 are listed in Table 3.

**Table 3:** The two-impulse values and its perilune altitudes.

No	①	②	③	④	⑤	⑥	⑦
$\Delta t$ (TU)	1.55	1.76	3.00	4.00	5.00	5.94	7.00
$\Delta v$ (km s^−1^)	4.2588	4.2509	4.3106	4.3455	4.3622	4.3695	4.3144
$h_{{{\text{prl}}}}$ (km)	2.6534	590.16	2349.1	2759.1	2880.1	3080.6	7054.0

**Figure 9:**
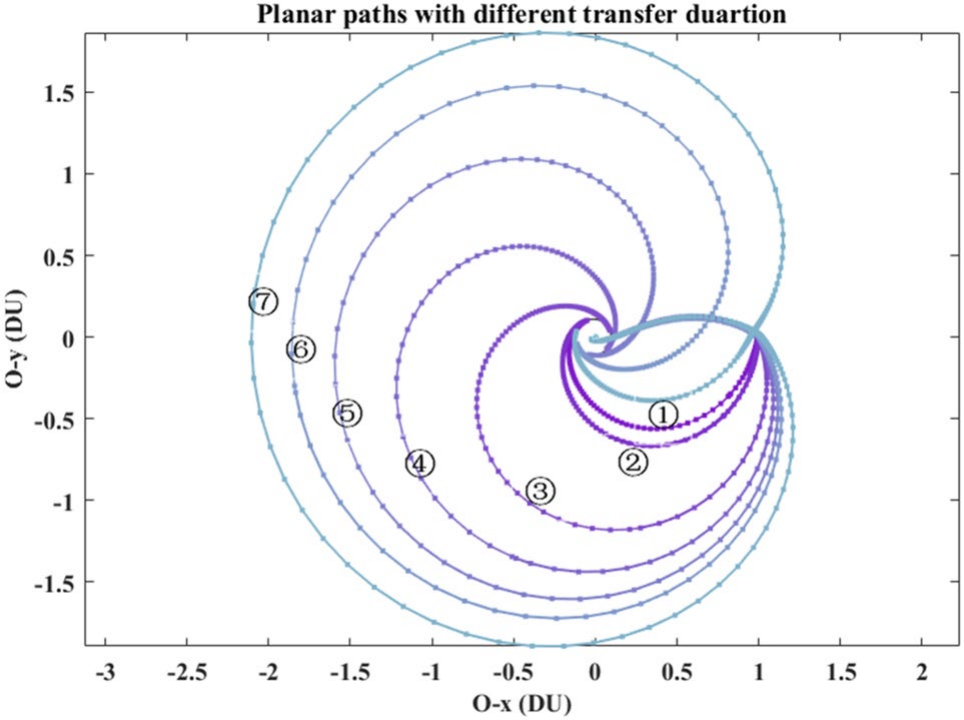
The planar paths in the overall view.

**Figure 10:**
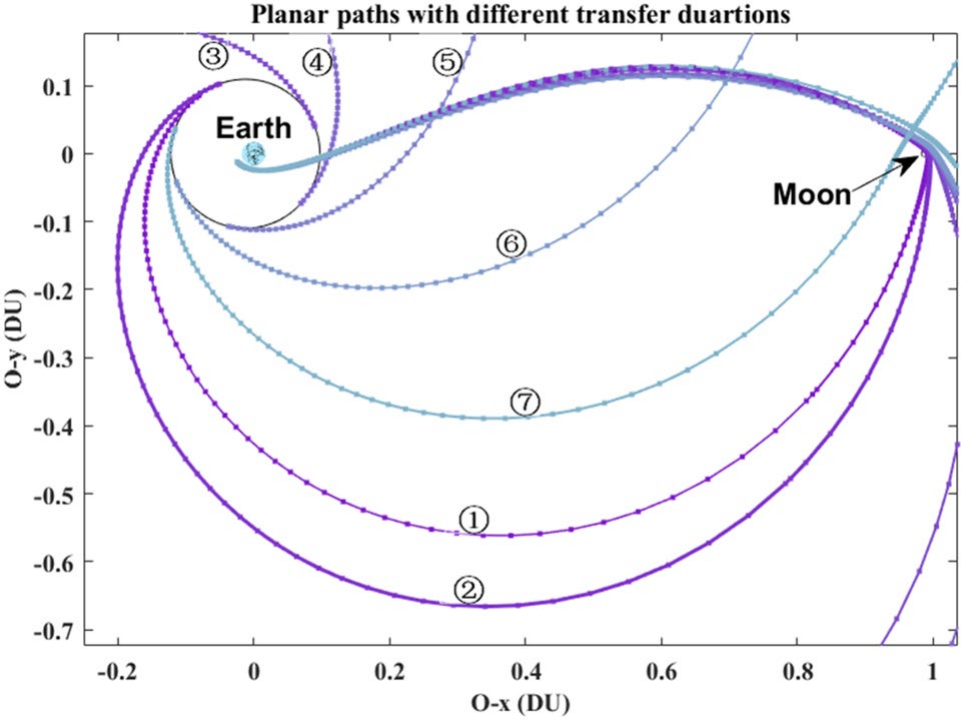
The detail paths in the Earth vicinity.

**Figure 11:**
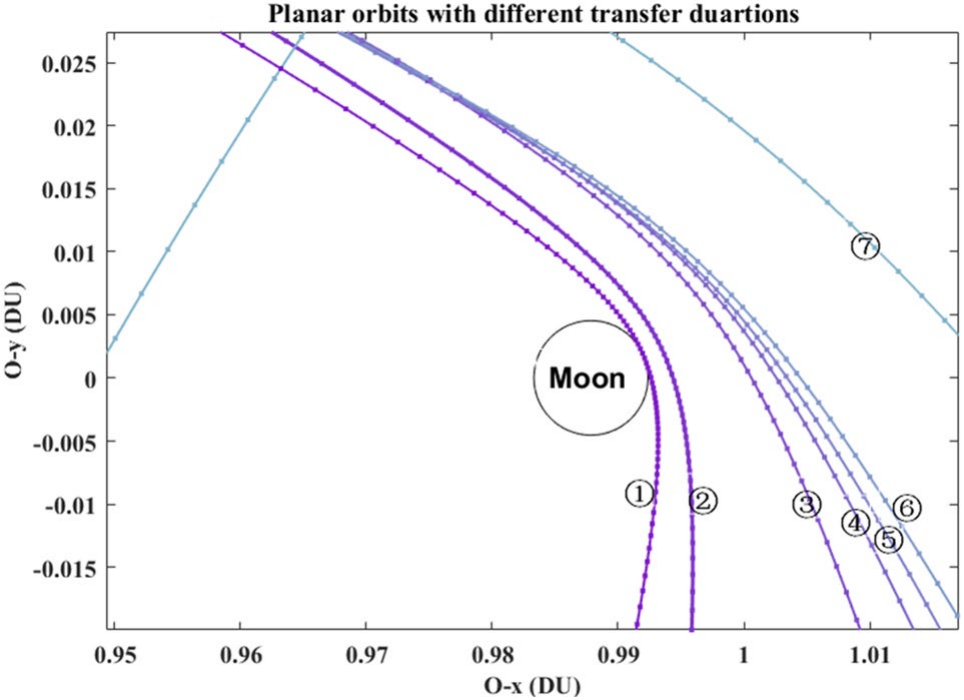
The detail paths in the Moon vicinity.

These seven planar paths with different transfer duration are plotted in Fig. 9. Its detailed paths in the Earth vicinity and in the Moon vicinity are shown in Fig. 10 and in Fig. 11, respectively. The *rkf-78* numerical integrator is used and its step size is $2 \times 10^{ - 2}$, so the markers of these paths are not uniform.

We all know that the Sun’s perturbation affects the transfer in the BR4BP. This time, the transfer duration $\Delta t$ is a constant, but the Sun’ azimuth plays the role of the continuation element to construct a frame as (12).

12 $${\text{Outer}}\left\{ \begin{array}{l}{\text{for }}{\theta _{{\text{mid}}}} = \theta _{{\text{mid}}}^{\min }:\theta _{{\text{mid}}}^{{\text{step}}}:\theta {{\text{mid}}}^{\max }\\{\text{Inner}}{\kern 1pt} {\kern 1pt} \left\{ \begin{array}{l}\user2{x} = \left[ {{\lambda _0},{V_0},{\lambda _{\text{f}}},{V_{\text{f}}}} \right]\\\min {\kern 1pt} {\kern 1pt} {\kern 1pt} {\kern 1pt} J = \Delta v\\{\text{s}}{\text{.}}{\kern 1pt} {\text{t}}{\text{.}}{\kern 1pt} {\kern 1pt} {\kern 1pt} {\kern 1pt} {\kern 1pt} {\kern 1pt} {\kern 1pt} \varepsilon = 0\end{array} \right.\\{\text{end}}\end{array} \right.$$

**Figure 12:**
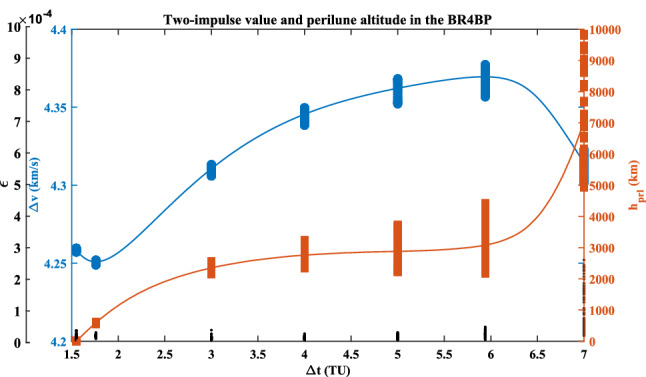
Two-impulse value and perilune altitude oscillate in the planar BR4BP.

Here, $\theta_{{{\text{mid}}}} = \omega_{s} \cdot t_{{{\text{mid}}}}$. $t_{{{\text{mid}}}}$ is the middle epoch of the transfer duration. When $\theta_{{{\text{mid}}}}$ changes from zero to $2{\uppi }$, the two-impulse value and the perilune altitude oscillate as plotted in Fig. 12.

**Figure 13:**
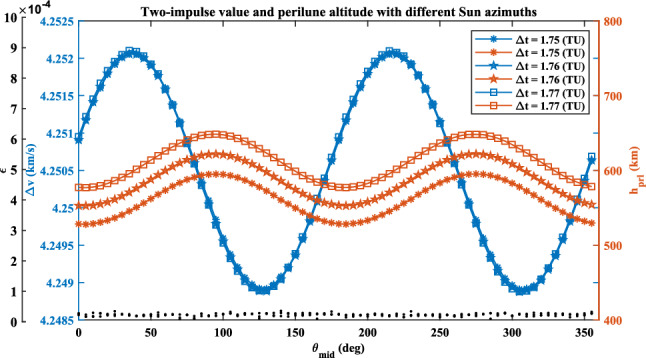
Two-impulse value and perilune altitude oscillate with different transfer duration.

The cumulative effect of the solar perturbation is obvious with the transfer duration increasing. In the case of $\Delta t = 1.76$ TU, $\Delta v$ has a range of [4.2489, 4.2521] ${\text{km}}\,{\text{s}}^{ - 1}$, $h_{{{\text{prl}}}}$ has a range of [552.61, 621.90] km. The transfer duration changes 0.01 TU (i.e., about 1 h), the value of $\Delta v$ is almost unchanged while the value of $h_{{{\text{prl}}}}$ has a difference of about 24.5 km. The detail difference is shown in Fig. 13.

## Properties in the spatial model

### Inclination changeable capacity via lunar swing-by

The original intention of this transfer via lunar swing-by is of changing the orbital inclination from a direct LEO to a retro-GEO orbit at the Earth-centric view. The orbital inclination changeable capacity via lunar swing-by is investigated in this Section. Based on the solutions in the planar model, the solution in the spatial model is easy to be calculated also using the continuation strategy as (13).

13 $${\text{Outer}}\left\{ {\begin{array}{*{20}l} {{\text{for }}\theta _{{\text{f}}} = \pi :\theta _{{\text{f}}}^{{{\text{step}}}} :2\pi \, {\& \&} \, \theta _{{\text{f}}} = \pi : - \theta _{{\text{f}}}^{{{\text{step}}}} :0} \hfill \\ {{\text{Inner}}\left\{ {\begin{array}{*{20}l} {\user2{x} = \left[ {\lambda _{0} ,\varphi _{0} ,V_{0} ,\theta _{0} ,\Delta t,\lambda _{{\text{f}}} ,\varphi _{{\text{f}}} ,V_{{\text{f}}} } \right]} \hfill \\ {\min \quad J = \Delta v} \hfill \\ {{\text{s.t.}}\quad \varepsilon = 0} \hfill \\ \end{array} } \right.} \hfill \\ {{\text{end}}} \hfill \\ \end{array} } \right.$$

**Figure 14:**
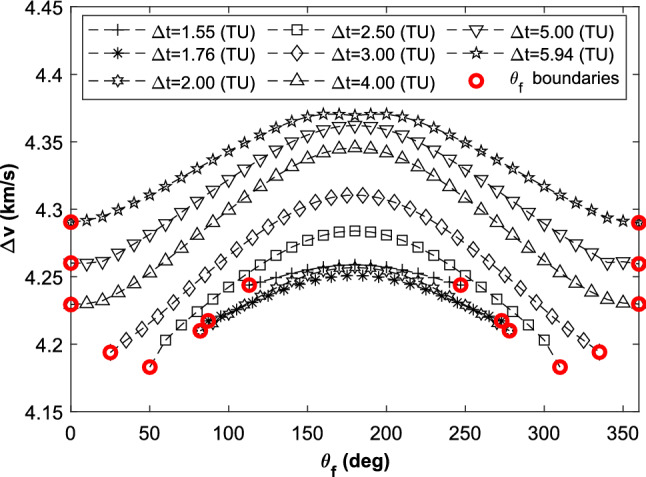
Inclination changeable capacity in the spatial CR3BP.

In the planar CR3BP, the value of $\theta_{{\text{f}}}$ is a constant of $\pi$. It’s the most important element which affects is inclination of inserting the retro-GEO. Here, it plays the role of the continuation element. Its value changes from $\pi$ to both zero and $2\pi$ in parallel. The solutions which satisfy the limit value of $\varepsilon \le 1 \times 10^{ - 4}$ are plotted as Fig. 14.

It displays the symmetrical features of the transfers in the spatial CR3BP model as proven by Ref.^19^. In order to express the relationship between the inclination changeable capacity and the transfer duration more clearly, the solutions of $\Delta t =$ 2.00 TU and $\Delta t =$ 2.50 TU are supplemented. When $\Delta t$ is more than 4.00 TU, just via lunar swing-by, the transfer could insert the retro-GEO with any inclination value. The detail values of the inclination changeable capacity with the transfer duration are listed in Table 4.

**Table 4:** The inclination changeable capacity and the transfer duration.

$\Delta t$ (TU)	1.55	1.76	2.00	2.50	3.00	4.00	5.00	5.94
$\theta_{{\text{f}}}^{\min }$ (°)	113	87	82	50	25	0	0	0
$\theta_{{\text{f}}}^{\max }$ (°)	247	273	278	310	335	360	360	360
$\Delta v$ (${\text{km}}\,{\text{s}}^{ - 1}$)	4.2438	4.2173	4.2099	4.1830	4.1939	4.2294	4.2601	4.2905

**Figure 15:**
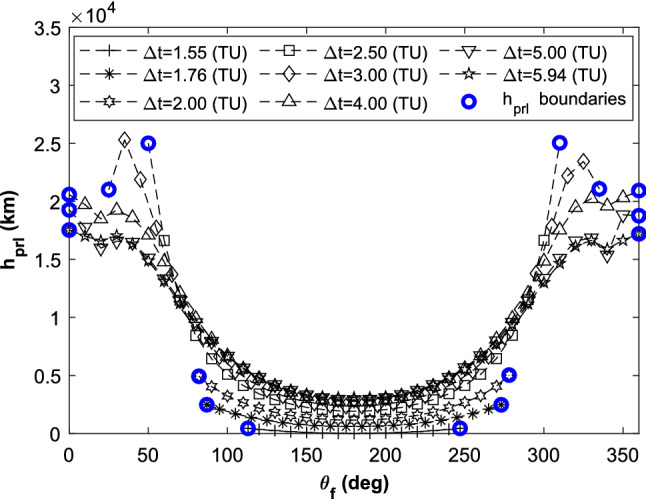
Perilune altitude changes in the spatial CR3BP.

In addition to this, the perilune altitudes of them are all more than zero. The validating data is plotted as shown in Fig. 15.

**Figure 16:**
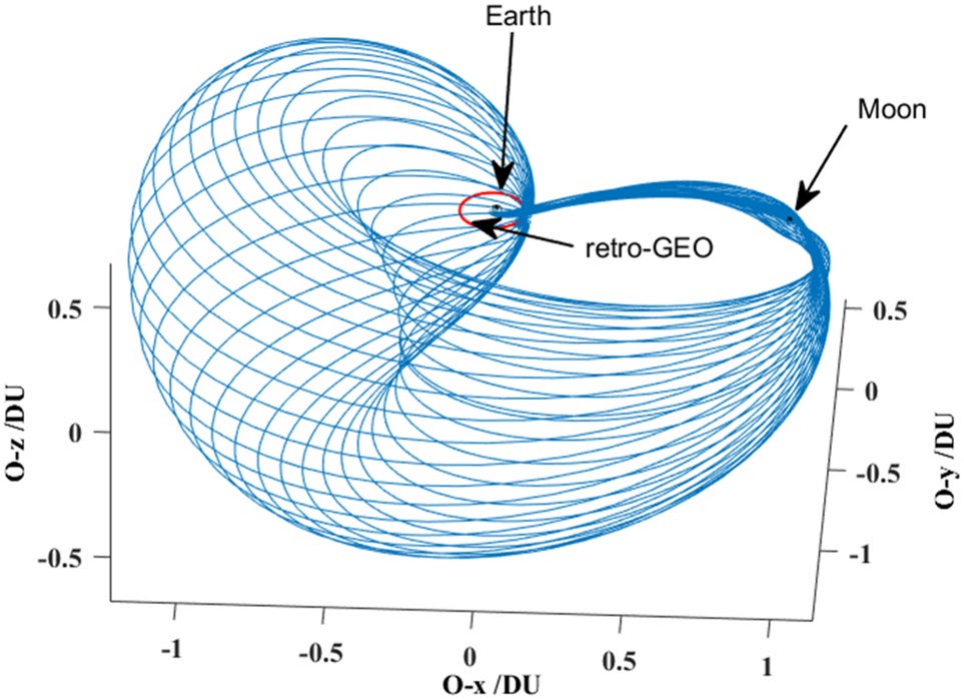
All the spatial paths of the case that $\Delta t{ = }$ 4.00 TU.

Here, all the spatial paths of the case that $\Delta t{ = }$ 4.00 TU are exhibited in Fig. 16. These spatial paths constitute an envelope.

### Sun-perturbed inclination changeable capacity

**Figure 17:**
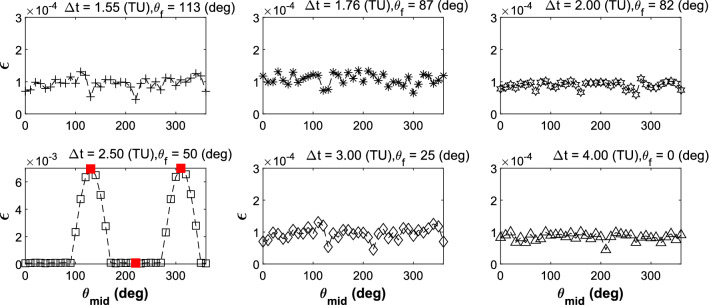
The value of $\varepsilon$ with different Sun’ azimuths.

Set $\Delta t$ and $\theta_{{\text{f}}}$ as constants in the spatial BR4BP, the Sun-perturbed inclination changeable capacity is exhibited by traversing all the Sun’s azimuths based on the solution in the planar CR4BP. The result is shown as Fig. 17.

**Figure 18:**
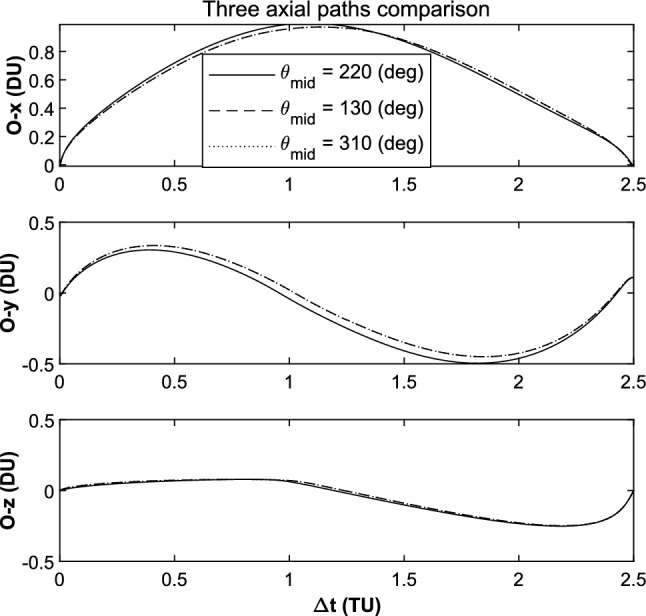
Three axial paths comparison.

In the most of the cases, $\varepsilon$ could approach the limited value of $1 \times 10^{ - 4}$. But in the case which $\Delta t{ = }$ 4.00 TU and $\theta_{{\text{f}}} { = }$ 50°, when $\theta_{{{\text{mid}}}}$ is near to 130° and 310°, the value of $\varepsilon$ reaches 6.9 $\times 10^{ - 3}$. The three axial paths comparison is shown in Fig. 18. It serves to show that the Sun’s perturbation difference occurs mainly in the $O{ - }xy$ planar.

## Conclusions

The properties of the transfers from an LEO to the retrograde-GEO via lunar swing-by both in CR3BP and in BR4BP are calculated and exhibited in this paper. The conclusions are drawn as follows:

1. The transfer constrained with the two tangential maneuvers for departing from an LEO and inserting into the retro-GEO exists just via once lunar swing-by.
2. The optimal two-impulse solution occurs when its transfer duration is 1.76 TU (i.e., 7.65 days), its Sun-perturbed range is [4.2489, 4.2521] ${\text{km}}\,{\text{s}}^{ - 1}$.
3. The orbital inclination changeable capacity is 93° when its two-impulse value is optimal. If its transfer duration is more than 4.00 TU (i.e., 17.59 days), it could insert the retro-GEO with any inclination via once lunar gravity assisted.
4. In the spatial BR4BP, the Sun’s perturbation does not affect this conclusion in most cases.

Extensive numerical calculations have been done in this work. The obtained results reveal some natural properties of this transfer and provide references to design a transfer using high-precision orbital dynamics model of deploying a monitor-satellite on the retro-GEO for debris-warning mission.

## References

[1] Li HN, Gao ZZ, Li JS (2013). Mathematical prototypes for collocating geostationary satellites. Sci. China Technol. Sci..

[2] Espinosa, S. A. Two new satellites now operational expand U.S. space situational awareness. Air Force Space Command Public Affairs. https://www.afspc.af.mil/News/Article-Display/Article/1310272/two-new-satellites-now-operational-expand-us-space-situational-awareness/ (2017). Accessed 26 April 2021.

[3] Berry, R. L. Launch window and trans-lunar orbit, lunar orbit, and trans-earth orbit planning and control for the Apollo 11 lunar landing mission. In *AIAA 8th Aerospace Sciences Meeting, New York. No.70-0024*. 10.2514/6.1970-24 (1970).

[4] Uesugi, K. Japanese fist double lunar swing-by mission "HITEN". In *41st Congress of the International Astronautical Federation, No. 1990-343*. 10.1016/0094-5765(91)90014-V (1990).

[5] Farquhar RW (2001). The flight of ISEE-3/ICE: Origins, mission history, and a legacy. J. Astronaut. Sci..

[6] Zeng GQ, Xi XN, Ren X (2000). A study on lunar swing-by technique. J. Astronaut..

[7] Luo ZF, Meng YH, Tang GJ (2010). Solution space analysis of double lunar-swingby periodic trajectory. Sci. China Technol. Sci..

[8] Oltrogge LD, Alfano S, Law C (2018). A comprehensive assessment of collision likelihood in geosynchronous earth orbit. Acta Astronaut..

[9] Oberg J (1984). Pearl harbor in space. Omni Mag..

[10] Kawase, S. Retrograde satellite for monitoring geosynchronous debris. In *16th International Symposium on Space Flight Dynamics, Pasadena, California, USA.* 3–7. http://home.k00.itscom.net/kawase/REF/2001-ISFD.pdf (2001). Accessed 26 April 2021.

[11] Kawase S (2010). Retrograde satellite to monitor overcrowded geosynchronous orbits. J-JSASS..

[12] Aravind R, Harsh S, Bandyopadhyay P (2012). Mission to retrograde geo-equatorial orbit (RGEO) using lunar swing-by. IEEE Aerosp. Conf. Proc..

[13] Li CL, Zuo W, Wen WB (2021). Overview of the Chang’e-4 mission: Opening the frontier of scientific exploration of the lunar far side. Space Sci. Rev..

[14] Topputo F (2013). On optimal two-impulse earth–moon transfers in a four-body model. Celest. Mech. Dyn. Astron..

[15] Szebehely V (1967). Theory of Orbits: The Restricted Problem of Three Bodies.

[16] Simó C, Gómez H, Jorba Á (1995). The Bicircular Model Near the Triangular Libration Points of the RTBP. From Newton to Chaos.

[17] Castelli R (2011). Nonlinear Dynamics of Complex Systems: Applications in Physical, Biological and Financial System.

[18] He BY, Shen HX (2020). Solution set calculation of the sun-perturbed optimal two-impulse trans-lunar orbits using continuation theory. Astrodynamics..

[19] Miele A, Mancuso S (2001). Optimal trajectories for earth–moon–earth flight. Acta Astronaut..

